# Structural insights into betaine aldehyde dehydrogenase (BADH2) from *Oryza sativa* explored by modeling and simulations

**DOI:** 10.1038/s41598-018-31204-z

**Published:** 2018-08-27

**Authors:** Apisara Baicharoen, Ranjit Vijayan, Prapasiri Pongprayoon

**Affiliations:** 1 Department of Chemistry, Faculty of Science, Kasetsart University, Chatuchak, Bangkok 10900 Thailand; 2 Department of Biology, College of Science, United Arab Emirates University, PO Box, 15551 Al Ain, Abu Dhabi United Arab Emirates; 3 Center for Advanced Studies in Nanotechnology for Chemical, Food and Agricultural Industries, KU Institute for Advanced Studies, Kasetsart University, Bangkok, 10900 Thailand; 4 Computational Biomodelling Laboratory for Agricultural Science and Technology (CBLAST), Kasetsart University, Bangkok, 10900 Thailand

## Abstract

Betaine aldehyde dehydrogenase 2 (BADH2) plays a key role in the accumulation of 2-acetyl-1-pyrroline (2AP), a fragrant compound in rice (*Oryza sativa*). BADH2 catalyses the oxidation of aminoaldehydes to carboxylic acids. An inactive BADH2 is known to promote fragrance in rice. The 3D structure and atomic level protein-ligand interactions are currently unknown. Here, the 3D dimeric structure of BADH2 was modeled using homology modeling. Furthermore, two 0.5 µs simulations were performed to explore the nature of BADH2 dimer structurally and dynamically. Each monomer comprises of 3 domains (substrate-binding, NAD^+^-binding, and oligomerization domains). The NAD^+^-binding domain is the most mobile. A scissor-like motion was observed between the monomers. Inside the binding pocket, N162 and E260 are tethered by strong hydrogen bonds to residues in close proximity. In contrast, the catalytic C294 is very mobile and interacts occasionally with N162. The flexibility of the nucleophilic C294 could facilitate the attack of free carbonyl on an aldehyde substrate. Key inter-subunit salt bridges contributing to dimerization were also identified. E487, D491, E492, K498, and K502 were found to form strong salt bridges with charged residues on the adjacent monomer. Specifically, the nearly permanent R430-E487 hydrogen bond (>90%) highlights its key role in dimer association. Structural and dynamic insights of BADH2 obtained here could play a role in the improvement of rice fragrance, which could lead to an enhancement in rice quality and market price.

## Introduction

Rice (*Oryza sativa*) has many flavours and textures that impact on its quality. Fragrance is a key factor that determines rice quality. Fragrant rice is gaining worldwide popularity among consumers. Its market price is much higher than common varieties of nonfragrant rice. Due to an increasing demand on fragrant rice worldwide, many studies have focussed on increasing yield of fragrant rice and retaining their fragrance^1,2^. 2-acetyl-1-pyrroline (2AP) has been found to be the most potent flavour compound that gives unique fragrance to jasmine and basmati rice^3^. Thus, the level of 2AP in rice serves as a key factor in determining the market price of fragrant rice.

Betaine aldehyde dehydrogenase 2 (BADH2) has been reported to play a role in the level of 2AP in rice^4,5^. The loss of BADH2 function accounts for the accumulation of 2AP resulting in an increase in rice fragrance^5^. BADH2 belongs to the aldehyde dehydrogenase (ALDH) family whose members include NAD(P)^+^-dependent enzymes catalysing the oxidation of many intermediate aldehydes to their corresponding carboxylic acids^6^. BADH2 oxidizes a broad range of aminoaldehydes (e.g. γ-aminobutyraldehyde (GAB-ald), 3-aminopropionaldehyde (AP-ald), and 4-N-trimethylaminobutyraldehyde (TMAB-ald)) in addition to its natural substrate, betaine aldehyde (Bet-ald)^7–9^. Furthermore, previous studies demonstrated that BADH2 catalyses the oxidation of GAB-ald more efficiently than Bet-ald^4,7^. Generally, BADH2 converts GAB-ald into GABA, but the absence or nonfunction of BADH2 results in the accumulation of GAB-ald which is subsequently converted to 2AP.

**Figure 1:**
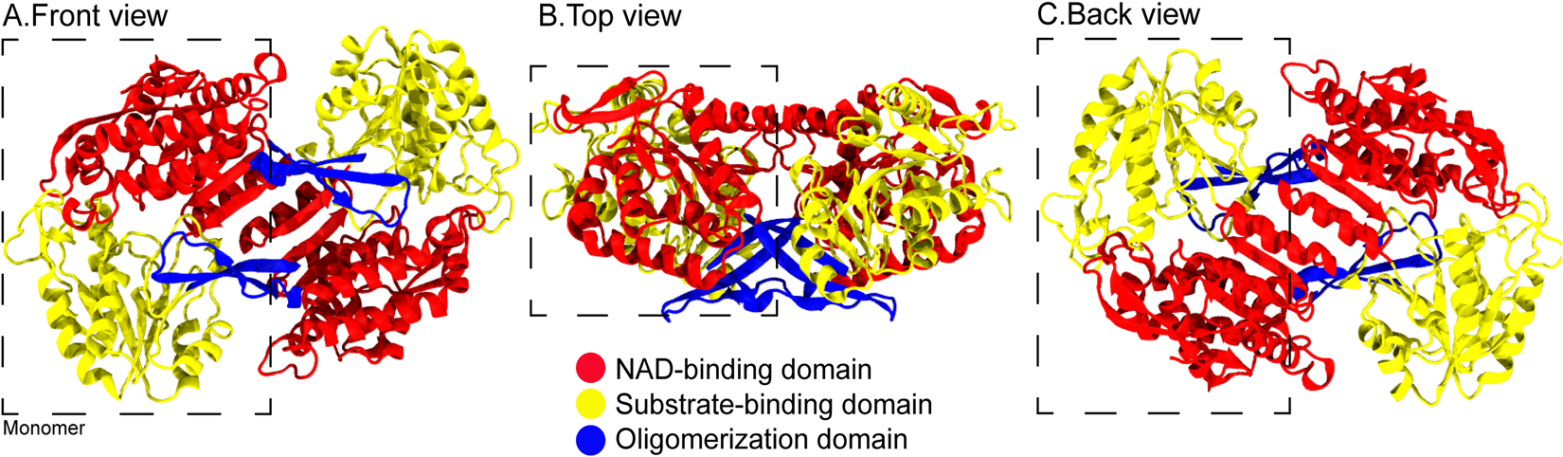
(**A**–**C**) Cartoon views of dimeric BADH2 with 3 domains (NAD^+^-binding domain in red, substrate-binding domain in yellow, and oligomerization domain in blue). The dashed line represents a monomer.

Currently, two crystal structures of AMADH from *Pisum sativum* are available^10^. Both are dimeric. Each AMADH unit contains a NAD^+^-binding domain, an oligomerization domain, and a substrate-binding or catalytic domain (Fig. 1). In the catalytic domain, the catalytic triad (N162, E260, and C294) is conserved in many species. Two BADH homologs, BADH1 and BADH2, from *Oryza sativa* share ~75% sequence similarity between the two and ~76% with AMADH. BADH1 was found to be mainly responsible for abiotic stress tolerance, while BADH2 contributes to rice aroma^4,8,11^. The level of BADH2 catalytic activity directly impacts the 2AP level^4,8^. Since the function of BADH2 is attributed to the aroma of rice, many studies have been devoted to understanding the nature of rice BADH2^11–18^. The ability to enhance rice aroma via controlling BADH2 activity can result in better rice quality and, importantly, higher market price.

BADH2 is found to be a dimer in plant^10^. For rice, each subunit (503 amino acids each) contains 3 domains (NAD^+^-binding, substrate-binding, and oligomerization domains) (Fig. 1). The long oligomerization domain latches two units together (coloured blue in Fig. 1). Previous simulation and mutagenesis studies have identified a few key residues (N162, Y163, M167, W170, E260, W288, S295, W420, C453, and W459) that are crucial for substrate binding and recognition^16–18^. Mainly, N162, E260, C294 were found to play a role in catalytic activity (Fig. 1C). C294 and E260 were involved in a key step of hemithioacetal-enzyme formation, while N162 helps stabilize an intermediate^16–18^. However, no clear atomic level evidence is available. Earlier mutagenesis work reported some key residues for BADH2 function and the dynamics of these residues was observed in a presence of bound substrate using molecular dynamics (MD) simulations^16–18^. However, such short simulations may not be sufficient to explore most of the dynamic and structural properties of BADH2. Thus, in this work, we employed 0.5 µs MD simulations to primarily observe the nature of native BADH2 in solution. Key structural and dynamic properties of BADH2 were also investigated. Structural insights of BADH2 obtained here will serve as a first step to understand the mechanism of fragrance production in rice which will be useful for future improvement to the fragrance of rice.

## Results

**Figure 2:**
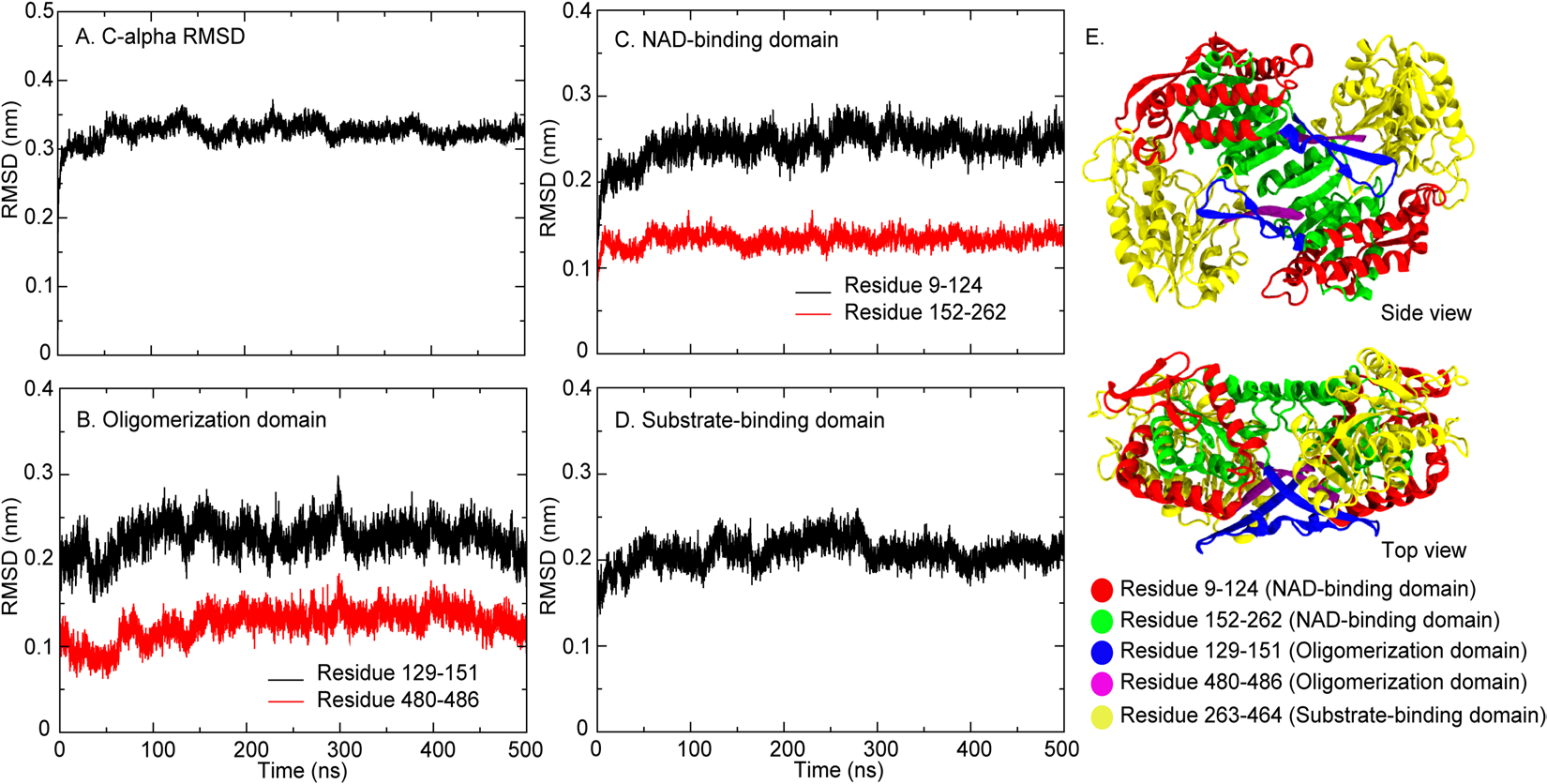
Average Cα RMSD is shown in (**A**). Average RMSDs of each domain are displayed in (**B**,**D**). The cartoon views of each monomer (M1 and M2) are shown in (**E**). Colours have been used to demarcate the regions used for the calculation of RMSDs in (**B**) to (**D**).

ClustalW webserver (https://embnet.vital-it.ch/software/ClustalW.html) was used to align and calculate the sequence similarity between BADH1 and BADH2 from rice, and AMADH from *P*. *sativum*^19^. Both BADH1 and BADH2 share 75.94% sequence similarity, while 76.54% sequence similarity was found between BADH2 and AMADH. Almost all the key residues in the substrate- and NAD^+^-binding domains, such as N162, E260, and C294, are conserved (Fig. S2 in Supplementary Information). The crystal structure of AMADH was used as the template to construct a 3D model of BADH2 using homology modelling. To explore structural changes and flexibility, we computed the root mean-square deviations (RMSDs) and fluctuations (RMSFs) of the three-dimensional BADH2 model in simulations. RMSD was calculated by comparing the movement of atoms with initial coordinates at t = 0. In general, Cα RMSD (Fig. 2A) indicated that the core protein structure became stable after 10 ns. Considering the individual domains, oligomerization and NAD^+^-binding domains are divided into 2 regions (residues 9–124 and 152–262 for NAD^+^-binding domain and residues 129–151 and 480–486 for oligomerization domain shown in Fig. 2E). Overall, the NAD^+^-binding domain seems to be the most mobile region due to the highest RMSD (~0.25 nm) (Fig. 2C). However, parts of these domains showed different degrees of structural fluctuations. Residues 152–262 in NAD^+^-binding domain displays less mobility than the other part in the same domain (Fig. 2C). Residues 480–486 in oligomerization domain also shows low mobility than the other region in the same domain due to the short length (only 6 amino acids in this region). The rest of the protein showed low protein flexibility (RMSDs of ~0.2 nm in Fig. 2B–D)

**Figure 3:**
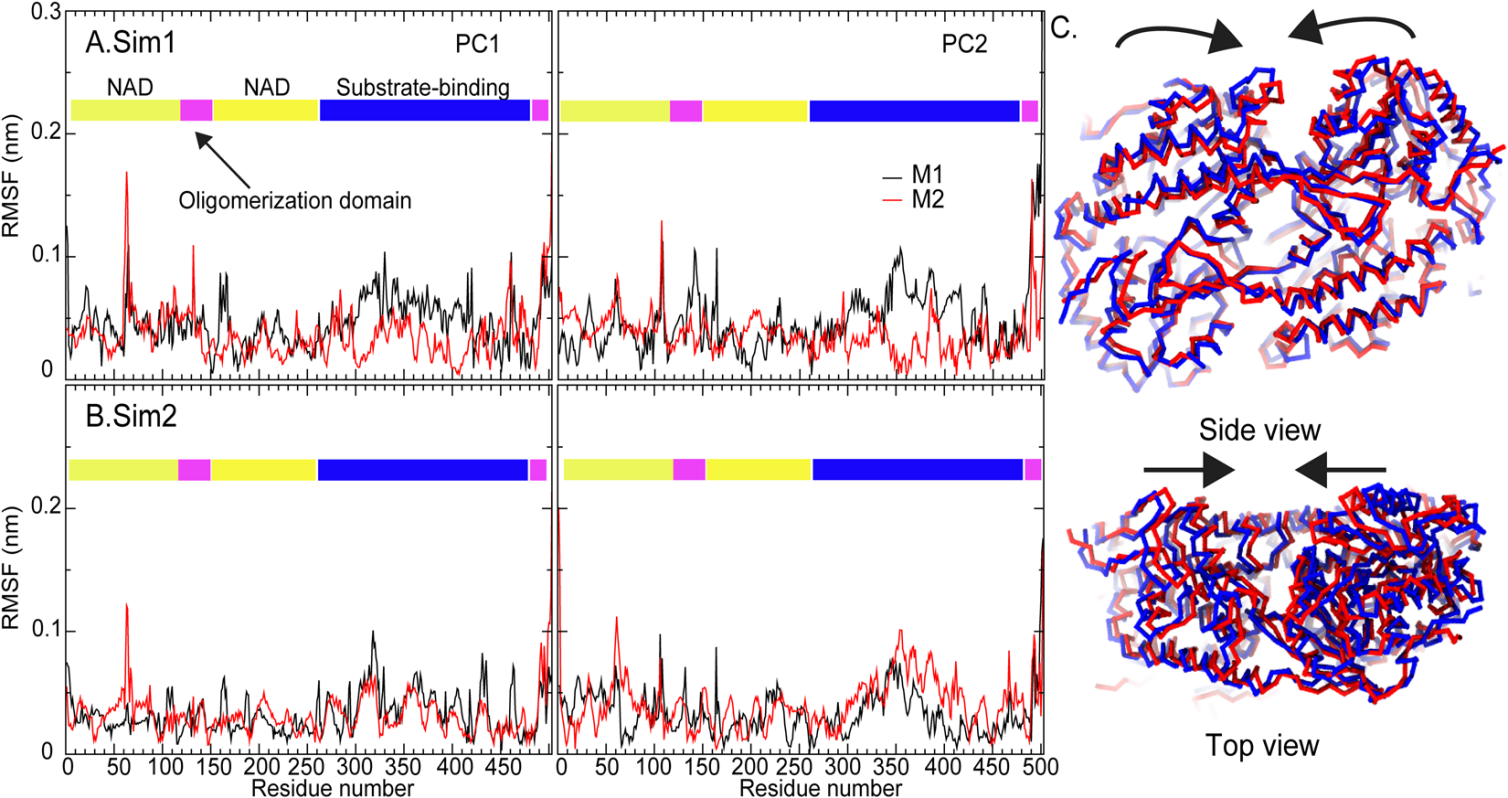
Cα RMSFs computed from Principal Component Analysis (PCA) of (**A**) Sim 1 and (**B**) Sim 2. The structures on the right represent the major motions of BADH2 obtained from the principal component 1 (PC1) from Sim 1. The blue trace shows an initial conformation, while the red displays the final orientation. Arrows indicate the direction of protein motion.

To explore protein dynamics, Principal Component Analysis (PCA) was performed on Cα atoms. This revealed that the first eigenvector (principal component 1 (PC1)) accounts for the major motions and there are indications that the second eigenvector (principal component 2 (PC2)) could also play a role (Fig. S3). Overall, the direction of main motions obtained from PC1 and PC2 are similar for both simulations (Fig. S4). The scissor-like motion is observed in all simulations (Figs 3C and S4). Mostly, both PC1 and PC2 give the same inward scissoring movement for both Sim 1 and Sim 2, except PC2 for Sim 2 where outward scissoring-like motion is observed (Fig. S4). Due to the sharp peak of RMSFs extracted from PCA, the NAD^+^-binding region (residues 50–100) adjacent to the oligomerization domain appeared to be flexible and contributed to the protein dynamics in both simulations (Fig. 3). This result agrees well with our RMSD calculation. It would appear that the high RMSDs and RMSFs observed indicate that NAD^+^-binding domain is the major contributor to protein motion. Besides this, the Cα atoms of the substrate-binding domains are also quite mobile (Fig. 3A,B). Furthermore, different degrees of backbone flexibilities observed from RMSFs reflect that dynamics of both monomers are not identical.

**Figure 4:**
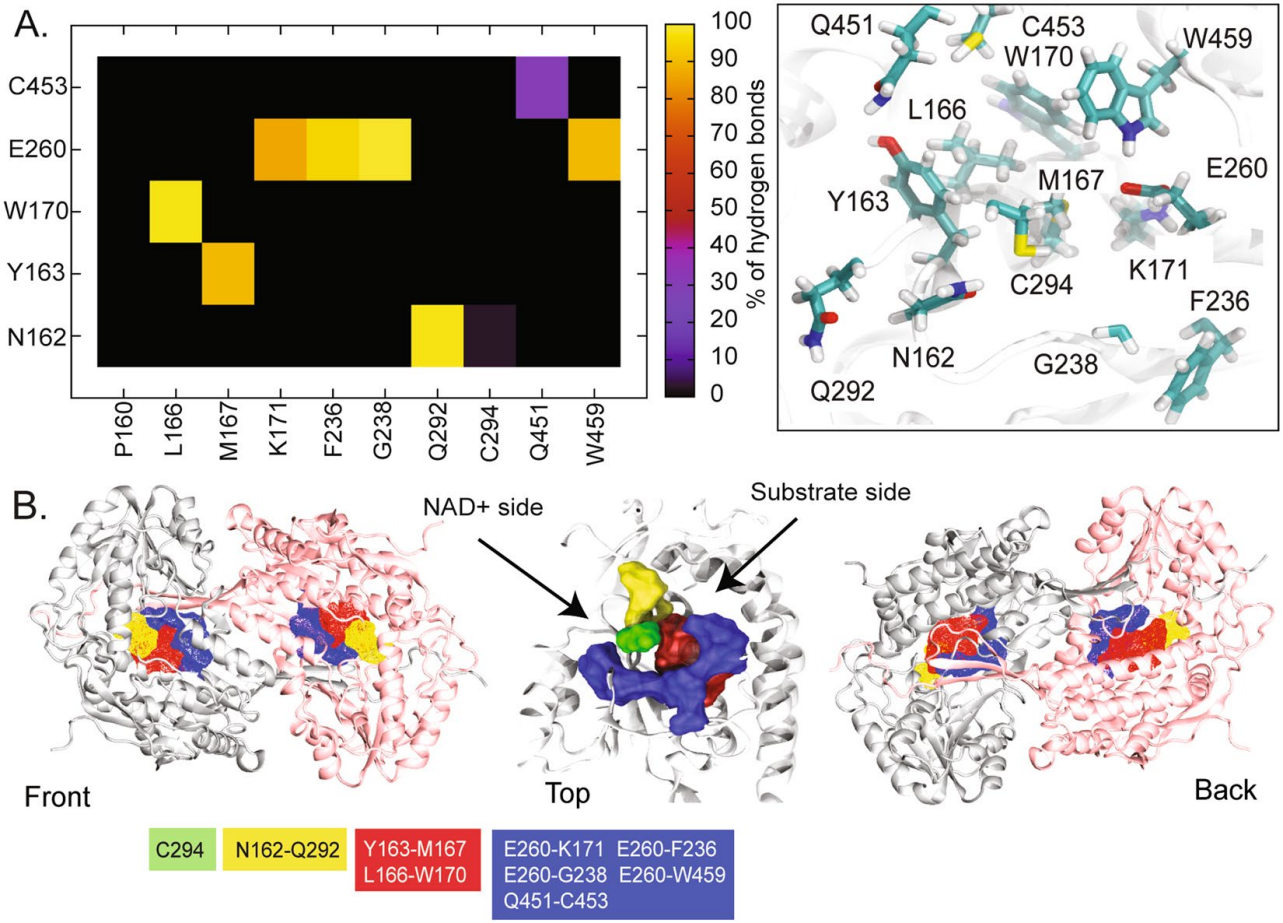
(**A**) The percentage of hydrogen bonds occurring throughout all simulations in the regions of substrate- and NAD^+^-binding domains. (**B**) Key amino acids inside the pocket are also shown in licorice format on the right. (**B**) Front and back views of dimeric BADH2 with red, yellow, and blue surfaces representing the location of each interaction network. The interactions in these regions are shown below in coloured boxes.

**Figure 5:**
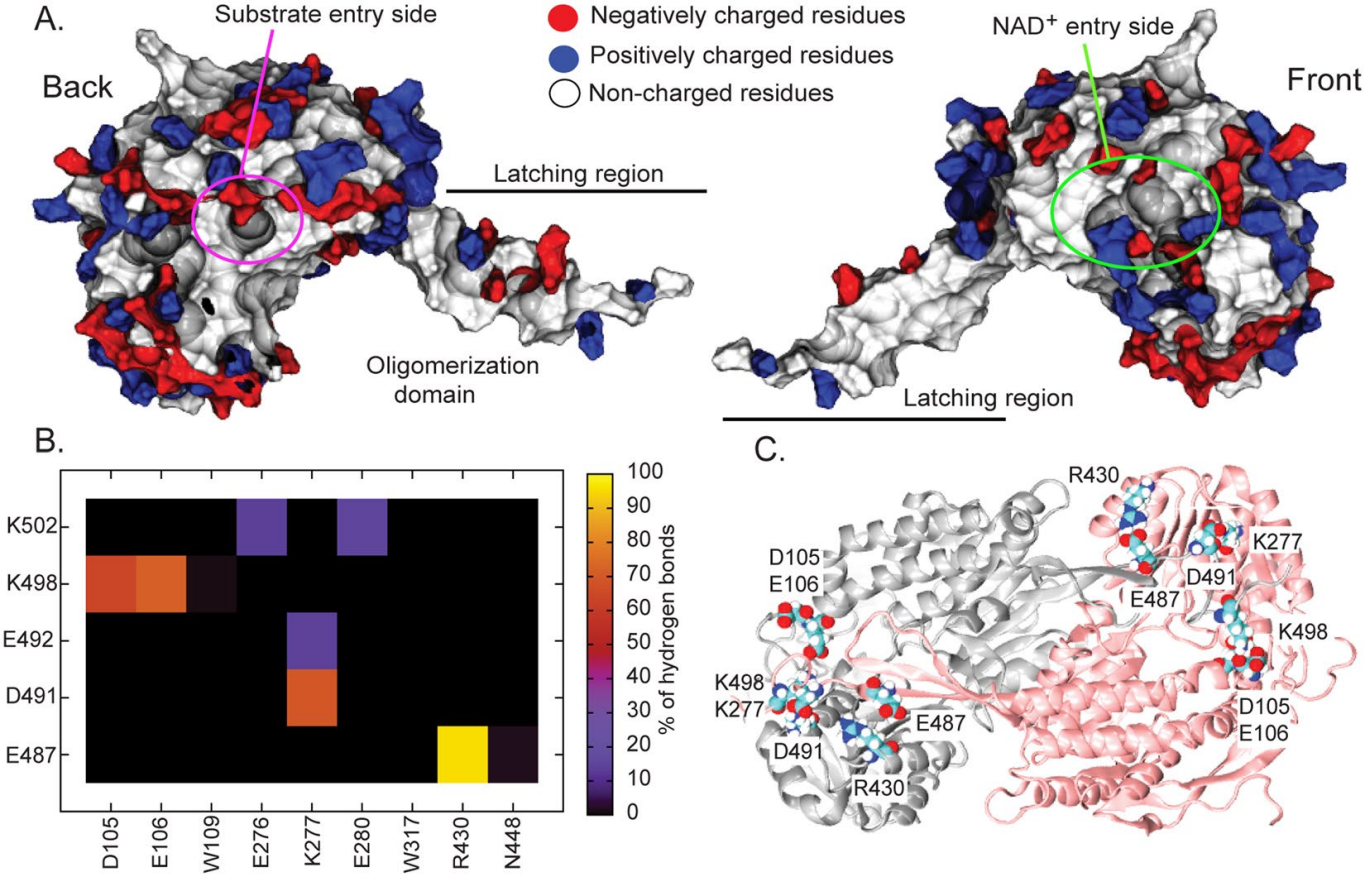
(**A**) Front and back surface views of BADH2 monomer. (**B**) The percentage of hydrogen bonds occurring throughout the simulations at the dimeric interface. (**C**) Dimeric BADH2 with key residues at the dimer interface.

To evaluate protein-protein and protein-solvent interactions, average hydrogen bonds between monomer and solvent was calculated. Comparable monomer-water and inter-monomeric hydrogen bond interactions were observed in both simulations (Table 1). Fig. 4A summarizes the interactions between key amino acids inside the binding pocket. Hydrogen bonds between key residues (N162, Y163, L166, W170, E260, C294, C453, W459) and their adjacent residues were computed. The position of the interaction network can be divided into 3 groups and these are shown as coloured surfaces in Fig. 4B. We define a channel at the opposite side of the oligomerization region as the “front” part, whereas the ‘back” refers to the side in contact with the oligomerization domain (Fig. 4B). The first group, N162 and C294, previously reported as key to catalytic activity, are located on the front part of the substrate- and NAD^+^-binding sites (green and yellow surfaces in Fig. 4B). Both N162 and C294 form different degrees of hydrogen bonds. C294 seems to weakly hydrogen bond with adjacent amino acids (<1% of hydrogen bonds with N162), whereas N162 forms a strong hydrogen bond with Q292 (>90%). Apparently, C294 seems to be flexible, whilst N162 is tethered by Q292 inside a pocket. The blue surface in Fig. 4B represents the second group (E260, C453, and 459) and the red one stands for the third group at the base (Y163, L166, W170) where strong hydrogen bonds between Y163-M167 and L166-W170 were observed (Fig. 4A). Additionally, one of the key catalytic residues, E260, located at the side of the binding sites formed a strong hydrogen-bonding network with many nearby residues. K171, F236, G238, and W459 were observed to form long lasting hydrogen bonds with E260 (>80% in Fig. 4A). Our results agree with a study that showed that E260 strongly interacts with W459, but BADH2 does not form any significant E260-E470 interaction as found in AMADH^10^. Besides this, a transient interaction between C453-Q451 was also captured. To date, both computational and experimental studies of ALDH family^10,20–23^ suggest the binding site of a substrate is at the back of protein, whereas the front area accommodates NAD^+^ binding. NAD^+^ was predicted to enter the binding pocket from the front and a substrate goes to the opposite opening (Figs 4B and 5A)^21^. The catalytic C294 is located in the middle of a cavity (green surface in Fig. 4B (center)). Residues Y163, L166, M167, and W170 are aligned at the base of the substrate-binding site (red surface in Fig. 4B (center)), whilst the N162-Q292 interaction drags the N162’s sidechain towards the NAD^+^ binding site. Upon substrate binding, C294 and E260 were reported to be involved in the formation of key hemithioacetal-enzyme intermediate in assistance with NAD^+^. The nucleophile C294 attacks an aldehyde carbonyl of the bound substrate and the deprotonation of a substrate is undertaken by E260. Unlike catalytic C294 and E260, N162 appears to play a minor role in stabilizing an intermediate by forming an oxyanion hole^24^. Our simulations demonstrate the high flexibility of C294 which supports the role of the nucleophile C294’s ability to attack a free carbonyl group of a bound substrate. On the contrary, N162 and E260 appear to be rigid due to strong interactions with their neighbours. Such interactions tethering N162 and E260 may help to shape a suitable environment for a catalytic activity.

**Table 1:** Average number of hydrogen bonds observed throughout each simulation.

	Sim 1		Sim 2	
	M1	M2	M1	M2
Water	946.28 ± 18.85	952.27 ± 18.96	955.13 ± 5.18.05	954.92 ± 18.42
M1:M2	42.21 ± 5.11		37.83 ± 5.68	

M1:M2 indicates the number of hydrogen bonds occurring between monomer 1 (M1) and monomer 2 (M2).

It has been reported that in ALDH, electrostatic attractions serve as a major player for dimeric association^25^. In the oligomerization domain, there is an accumulation of charged residues at the base and some are distributed along the latching area (Fig. 5A). In the latching region, side chains of charged residues appear to point towards the bulk, while non-charged amino acids seem to line the dimer-dimer interface (Fig. 5A). Here, key electrostatic interactions on the latching region have been identified for the first time. Figure 5B,C illustrates key inter-subunit salt bridges between dimers and their locations. Charged residues at the C-terminus seem to play a role in dimer assembly. Permanent R430-E487 hydrogen bond (>90%) highlights its key role in dimer association (Fig. 5B). Moreover, not only moderate hydrogen bonds of K498-D105, K498-E106, D491-K277 (~50–60%), but also transient interactions of K277-E492, K502-E276, and K502-E280 (<40%) have been captured (Fig. 5B,C). Despite the fact that uncharged dimer contacts have been observed, inter-subunit salt bridges have also been observed between charged residues (E487, D491, E492, K498, and K502) on the latching loop between adjacent monomers. These act as clips that hold the dimer together.

## Discussion

In this study, the dynamic properties of dimeric BADH2 were investigated. The NAD^+^-binding domain was found to be very mobile and contributes to most of the dynamics of BADH2. Key interactions in the ligand binding pocket have also been revealed. N162 and E260 form strong hydrogen bonds with nearby residues resulting in a rigid conformation. The strong interaction network may help to shape a suitable cavity size and dimension that fits both substrate and cofactor. In contrast, C294 was found to be very mobile inside the pocket. It weakly interacted with N162. Its flexibility could facilitate the attack of carbonyl on an aldehyde substrate. The mobility of the catalytic cysteine is similar to that of other ALDHs from previous MD and QM/M studies^22,23,26^. Furthermore, key inter-subunit salt bridges contributing to dimerization have been identified for the first time. Although the dimer interface is lined by uncharged residues, both electronegative and electropositive residues on the latching region - E487, D491, E492, K498, and K502 - have been found to form strong interactions with charged residues on the other monomer. Such charged residues seem to play a role in dimer formation and stability.

Within the ALDH family, research interest has been drawn to protein-ligand binding affinities and catalytic mechanisms^16,22,23,26,27^. The highly conserved sequence in the catalytic pocket defines similar residues for catalysis. The overall picture of the reaction mechanism seems to be well-defined. However, not many studies have been devoted to protein structure and dynamics. Our work appears to be the first to address this gap. Each ALDH protein must have a clearly differentiated mechanism so as to obtain its unique function. Thus, understanding the ligand-protein interactions, dynamics, and detailed enzymatic mechanism of each protein remain crucial. An insight into the structure and function of BADH2 will be useful for future improvement of rice fragrance, which could lead to an enhancement of rice quality and market price.

## Methods

The sequence of BADH2 was obtained from Uniprot (Entry: O24174). A BLAST search based on the protein data bank revealed that the structures most similar to BADH2 was the 3-dimensional structure of aminoaldehyde dehydrogenase (AMADH) from *Pisum sativum* (PDB codes: 3IWK and 3IWJ) with a sequence similarity of 76.54%. The 3D model of dimeric BADH2 (503 amino acids each) was built by homology modelling using MODELLER (version 9.17)^28^. The quality of the model was checked by Ramachandran structure validation using RAMPAGE (http://mordred.bioc.cam.ac.uk/~rapper/rampage.php)^29^. The model developed had residues in favored (96.6%), allowed (2.8%) and outlier (0.6%) regions (Fig. S1 in Supplementary Information (SI)). Structures with residues over 90% in the favored region display a good structure quality as reported in both computational and experimental studies^30–33^. In this work, M1 refers to monomer 1 and M2 is for monomer 2. The protonation states of charged amino acids were set as that at physiological pH.

We employed GROMACS 5.0 package (www.gromacs.org)^34^ with Amberff99SB-ILDN force field. Particle mesh Ewald (PME)^35^ method with a Fourier spacing of 0.12 nm and a short range cut-off of 1 nm was used for electrostatic treatment. The system contained dimeric BADH2 and 86,416 TIP3P water molecules. Counter ions were added to neutralize the simulation system. Energy minimization of 1000 steps was performed to remove bad contacts using steepest descent algorithm followed by 10 ns of equilibration where the protein atoms were restrained with a force constant of 1000 kJ mol^−1^ nm^−2^. Next, two 0.5 µs production runs were performed. The simulations were conducted in the constant number of particles, pressure, and temperature (NPT) ensemble. The Berendsen algorithm at 1 bar with a coupling constant τ_p_ = 1 ps was used for pressure coupling. The temperature of the protein and solution were coupled separately using the v-rescale thermostat^36^ at 300 K with a coupling constant τ_t_ = 0.1 ps. The time step of 2 fs was used for integration. The coordinates were recorded every 2 ps.

Simulations trajectories were analyzed using GROMACS tools and in-house code. RMSD and RMSF calculations were performed using the initial structure from each of the two simulations. The hydrogen bonds were computed using g_hbond with default parameters (The hydrogen-donor-acceptor cutoff angle is 30° and the cutoff radius (X-acceptor) is 0.35 nm). VMD was used for visualization and generation of images^37^.

## Electronic supplementary material

Supplementary information
