# Supplementary material for: Structural insights into betaine aldehyde dehydrogenase (BADH2) from Oryza sativa explored by modeling and simulations
Source: Sci Rep. 2018 Aug 27;8:12892. doi: 10.1038/s41598-018-31204-z (PMC6110774; doi:10.1038/s41598-018-31204-z)
Supplement: Supplementary file 1 — Supplementary information [file 41598_2018_31204_MOESM1_ESM.pdf]

Supplementary Information

Structural insights into betaine aldehyde dehydrogenase (BADH2) from *Oryza sativa* explored by modeling and simulations

Apisara Baicharoen[1], Ranjit Vijayan[2*], Prapasiri Pongprayoon[1,3,4*]

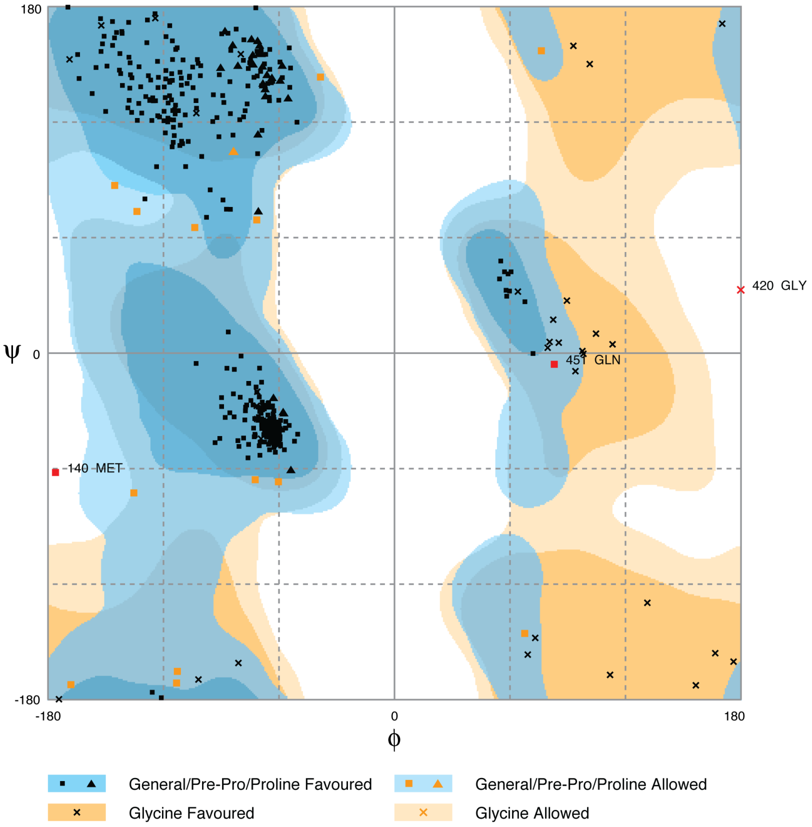

Figure S1 Ramachandran plot of BADH2 calculated form RAMPAGE [1]. The allowed region is shown as dark blue colour. The glycine favoured and allowed regions are in pale orange. The disallowed region is in white.

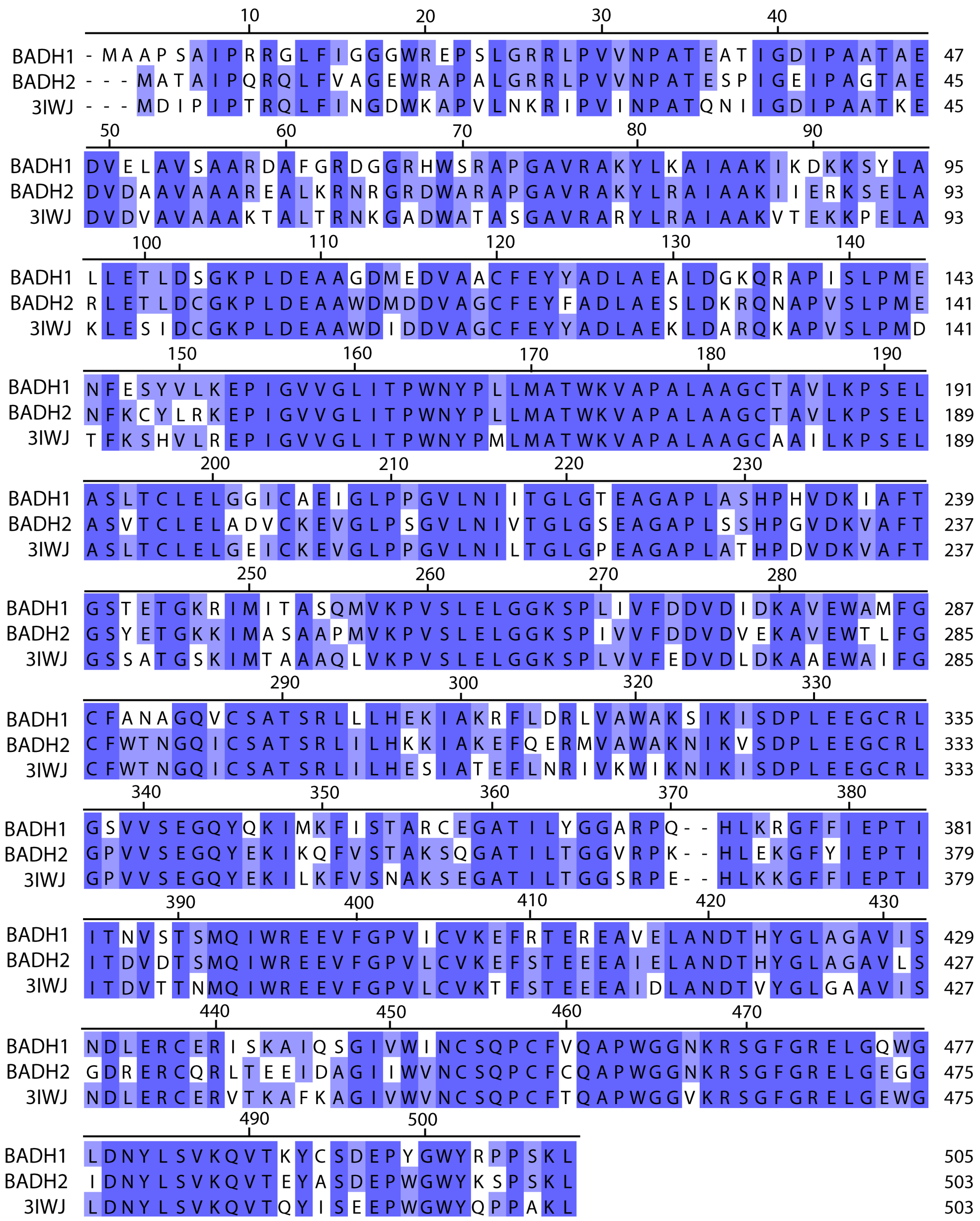

Figure S2 Sequence alignments among BADH1, BADH2, and AMADH (PDB code: 3IWJ) generated by JalView software [2]

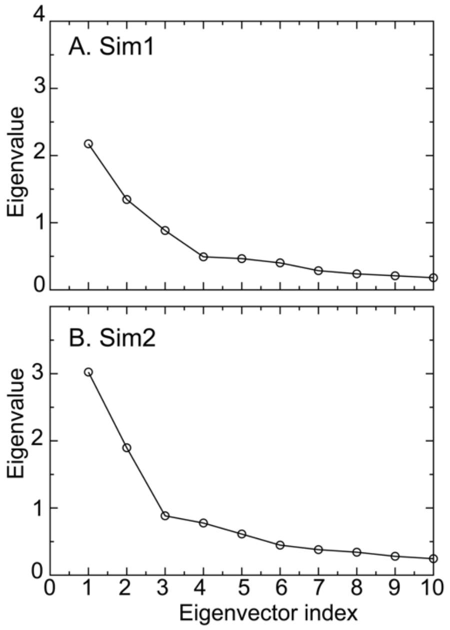

Figure S3 Eigenvalues of each simulations (sim1 and sim2) as a function of eigenvectors. The eigenvalues are derived from principal component analysis of trajectories of Cα atoms.

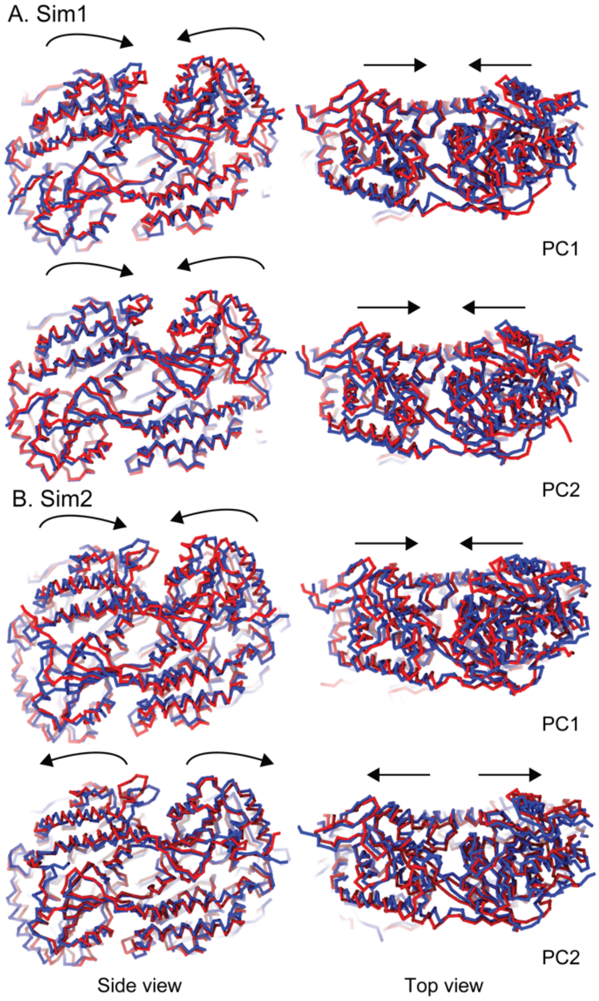

Figure S4 Protein motions obtained from Principal Component Analysis (PCA) using only Cα atoms. The major motions obtained from principle components 1 (PC1) and 2 (PC2) are shown. (A) and (B) stand for sim1 and sim2, respectively. The black arrow represents the direction of each motion. The blue and red colours show the displacement from t=0 to t=500 ns.

References

1 Lovell, S. C. et al. Structure validation by Calpha geometry: phi,psi and Cbeta deviation. *Proteins* **50**, 437-450, doi:10.1002/prot.10286 (2003).

2 Clamp, M., Cuff, J., Searle, S. M. & Barton, G. J. The Jalview Java alignment editor. *Bioinformatics* **20**, 426-427, doi:10.1093/bioinformatics/btg430 (2004).
